# Editorial: Editor’s challenge: Mohamed Rahouma - how can we optimize the management and treatment of cardiac tumors and cardiac complications of cancer?

**DOI:** 10.3389/fonc.2023.1271452

**Published:** 2023-08-30

**Authors:** Mohamed Rahouma, Massimo Baudo, Magdy M. El-Sayed Ahmed

**Affiliations:** ^1^ Cardiothoracic Surgery Departments, Weill Cornell Medicine, New York, NY, United States; ^2^ Surgical Oncology Department, National Cancer Institute, Cairo University, Cairo, Egypt; ^3^ Cardiac Surgery Department, Spedali Civili di Brescia, University of Brescia, Brescia, Italy; ^4^ Cardiothoracic Surgery Department, Mayo Clinic, Jacksonville, FL, United States; ^5^ Department of Surgery, Zagazig University Faculty of Medicine, Zagazig, Egypt

**Keywords:** cardiac tumors, cancer, atrial myxoma, cardiotoxicity, cardiac complications

Cardiac tumors are a heterogenous group of neoplasm that can be primary (benign or malignant), or secondary (metastatic) (1). Understanding their epidemiology on a large scale would significantly benefit treatment algorithms, but to this regard current evidence is scant. Interest in cardiac tumors has been progressively growing, as they are now considered curable conditions that can be detected and surgically excised at an early stage (2).

Cancer global survival rate has been steadily increasing due to diagnosis and treatment improvements. Nevertheless, cancer treatment is often associated with adverse cardiac reactions that may also affect the heart. This is also known as cancer therapy-induced cardiotoxicity and may negatively impact cancer survivors’ prognosis (3). Therefore, one of the aims of Cardio-oncology is to study the cardiovascular effects of cancer and its treatment.

This Research Topic in Frontiers in Oncology comes to shed light on the management and treatment of cardiac tumors and cardiac complications of cancer.

Two papers focused on cardiac tumors (Rahouma et al., Hao et al.). An interesting analysis of the Surveillance, Epidemiology, and End Results (SEER) database was conducted on the geographical variation in the United States of malignant cardiac tumors. Despite baseline characteristics differences of cardiac tumor patients exist between regions, none of the geographic areas showed a significant disparity in long-term survival. Surgery and chemotherapy were confirmed to be protective factors for mortality (Rahouma et al.).

A rare case of left atrial myxoma in an adolescents with hyperthyroidism was described (Hao et al.). This case report should remind physicians that left atrial myxoma can occur also in adolescents and early diagnosis and treatment of such diseases may prevent more catastrophic consequences. Possible features include recurrent rash and cerebral embolism.

Zacek et al. described their single-center experience, over a period of 30 years, of 37 nephrectomies and level IV (right atrial) thrombectomy with deep hypothermic circulatory arrest. The Authors have shown, despite the surgical complexity, how it can be performed safely with a generally uneventful postoperative course. The 5-years and median estimated cancer-related survival were 51% and 5.7 years (95% confidence interval (CI): 2.0–5.8 years) respectively.

The final two papers have focused on cancer therapy-induced cardiotoxicity. Cheng et al. have reviewed the discovery, biogenesis and general function of circular RNA. They also outlined significant research that underscores the therapeutic prospects of circRNAs in addressing cardiotoxicity and elucidated the hypothesized mechanisms underlying their biological effects. This unveiled the immense potential of circRNAs for both therapy and diagnosis. Further research in models and trials is warranted. Ye et al. assessed cardiac magnetic resonance (CMR) markers of myocardial fibrosis in gynecologic cancer patients with low cardiovascular risk who undergo chemotherapy. Sixty-eight patients and 30 controls were enrolled in this study. Compared to the control group, patients exhibited higher extracellular volume fraction and global longitudinal strain. The number of chemotherapy cycles correlated with increased extracellular volume fraction and decreased intracellular mass indexed. From this study, it emerges that patients with gynecologic cancer and low cardiovascular risk who undergo chemotherapy have diffuse extracellular volume expansion, which occurs with the increase of chemotherapy cycles. Myocyte loss may be part of the mechanism in patients with a higher chemotherapy load.

The brief overview of the articles included in this Research Topic provides interesting updates regarding the treatment of cardiac neoplasms and cardiac complications of cancer. There is a correlation between cancer and cardiovascular disease as they share risk factors. Additionally, the relationship between the two is becoming more complex (4). Therefore, it is crucial to foster collaborative research and care between cardiologists, and oncologists to gain a clearer understanding of the interactions between treatments, pathogenesis, and other aspects of heart and cancer.

## Author contributions

MR: Conceptualization, Project administration, Supervision, Writing – original draft, Writing – review & editing. MB: Conceptualization, Project administration, Visualization, Writing – original draft, Writing – review & editing. MA: Conceptualization, Project administration, Supervision, Writing – review & editing.

## References

[1] Rahouma M Arisha MJ Elmously A El-Sayed Ahmed MM Spadaccio C Mehta K. Cardiac tumors prevalence and mortality: A systematic review and meta-analysis. Int J Surg Lond Engl (2020) 76:178–89. doi: 10.1016/j.ijsu.2020.02.039 32169566

[2] Poterucha TJ Kochav J O’Connor DS Rosner GF. Cardiac tumors: clinical presentation, diagnosis, and management. Curr Treat Options Oncol (2019) 20:66. doi: 10.1007/s11864-019-0662-1 31250250

[3] Minotti G Menna P Salvatorelli E Cairo G Gianni L. Anthracyclines: molecular advances and pharmacologic developments in antitumor activity and cardiotoxicity. Pharmacol Rev (2004) 56:185–229. doi: 10.1124/pr.56.2.6 15169927

[4] Boudoulas KD Triposkiadis F Gumina R Addison D Iliescu C Boudoulas H. Cardiovascular disease, cancer, and multimorbidity interactions: clinical implications. Cardiology (2022) 147:196–206. doi: 10.1159/000521680 34986484

